# Nonuniform Chemical Passivation Dynamics in Monolayer MoS_2_ from Real-Time Photoluminescence Imaging

**DOI:** 10.1021/acs.jpclett.6c00839

**Published:** 2026-05-22

**Authors:** Juhwan Lim, Jiho Han, Nicolas Gauriot, Zhaojun Li, Jung-In Lee, Christoph Schnedermann, Manish Chhowalla, Akshay Rao

**Affiliations:** † Cavendish Laboratory, 2152 University of Cambridge, JJ Thomson Avenue, Cambridge CB3 0HE, United Kingdom; ‡ Department of Materials Science and Metallurgy, 2152 University of Cambridge, 27 Charles Babbage Road, Cambridge CB3 0FS, United Kingdom; § Solid State Physics, Department of Materials Science and Engineering, Uppsala University, P.O. Box 35, SE-751 05 Uppsala, Sweden

## Abstract

Postchemical treatment is a major strategy for improving the optical and electronic performance of two-dimensional transition-metal dichalcogenides (TMDs), where surface defects and unintentional doping limit radiative recombination. Despite its broad application, the passivation dynamics in both time and space remain insufficiently understood. Here, we employ real-time, spatially resolved photoluminescence (PL) imaging to track the evolution of PL emission from monolayer MoS_2_ during the Li-TFSI treatment. The PL intensity increases gradually over 4.7 min, indicating reaction-limited passivation kinetics. At the same time, the spatial variation in PL becomes more pronounced, revealing position-dependent reactivity and heterogeneous treatment across the flake. Pixel-level PL statistics compared with noise-based simulations confirm that this broadening originates from the intrinsic spatial heterogeneity of the material. These results demonstrate that chemical treatment enhances radiative recombination while simultaneously amplifying pre-existing spatial differences, establishing real-time PL imaging as a sensitive approach to probing heterogeneous surface reactions in 2D semiconductors.

## Graphical abstract

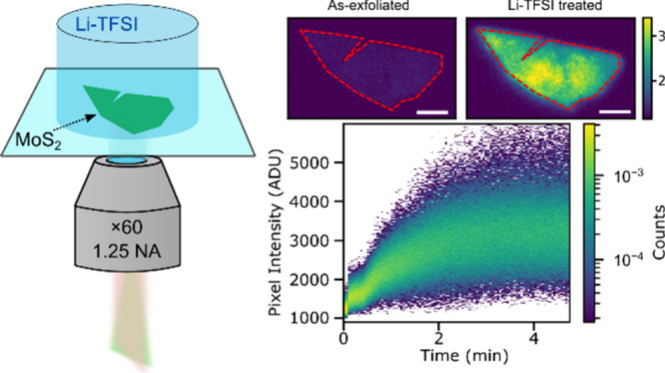

Radiative recombination is a fundamental optical process in semiconducting materials and reflects the intrinsic electronic structure, doping, and defect profile through the dynamics of excitons.^1,2^ In monolayer two-dimensional transition-metal dichalcogenides (TMD), radiative emission is strongly suppressed by structural defects and unintentional doping, both of which introduce nonradiative pathways and lower the photoluminescence quantum yield (PLQY) of as-prepared samples.^3–5^ Numerous studies have focused on enhancing the radiative recombination yield, typically observed as increased photoluminescence (PL) intensity, not only to improve optoelectronic device performance but also to exploit PL as a sensitive probe of the material quality, excitonic behavior, doping nature, and defect states.^6–10^ Postsynthetic chemical treatments have emerged as an effective approach for mitigating nonradiative recombination by neutralizing unintentional doping and passivating defects, thereby enhancing PL intensity.^2,11–14^ Treatments using p-dopants or superacids such as hydrogen bis­(trifluoromethane) sulfonimide (H-TFSI) and lithium salts such as lithium bis­(trifluoromethanesulfonyl)­imide (Li-TFSI) substantially increase the population of neutral excitons and enhance PL in both exfoliated and CVD-grown TMD monolayers.^13–18^ Despite these advances, the spatial and temporal dynamics of chemical passivation, particularly how uniformly the treatment proceeds across a monolayer and how the PL evolves in time, remain insufficiently understood.

Here, we investigate the kinetics and heterogeneity of Li-TFSI treatment in mechanically exfoliated monolayer MoS_2_ using *in situ* optical microscopy, which tracks reaction mechanisms and enables a statistically robust analysis of their spatiotemporal distributions.^19,20^ As an archetypal group-6 semiconducting TMD, MoS_2_ stands out as one of the most promising candidates for ultrathin semiconductors.^21^ Its excitonic emission is highly sensitive to local charge density, the exciton/trion ratio, and defect chemistry and has been shown to enable optical monitoring of redox and catalytic processes, substrate effects, and defect dynamics.^22–26^ To resolve the spatial and temporal features of Li-TFSI passivation of MoS_2_, we use a real-time PL imaging platform that records the emission intensity profile of a flake as the chemical treatment proceeds. Monitoring the entire monolayer simultaneously allows us to examine how the reaction progresses and how different regions of the flake respond, which depends on their defect environment and local accessibility to Li cation adsorption. The pixel-to-pixel intensity variation is analyzed using a histogram approach and visualized with percentile-based mapping. We quantify the role of measurement-related broadening and conclude that spatial heterogeneity exists and increases as the PL enhancement progresses. Furthermore, we show that this spatial structure is set at the beginning of enhancement. This study provides the level of detail needed to interrogate heterogeneous passivation pathways and establishes how chemical treatments interact with the intrinsic defect structure of two-dimensional semiconductors.

Figure 1A shows a schematic of the home-built photoluminescence (PL) microscopy setup used for monitoring the PL enhancement of MoS_2_ during Li-TFSI treatment. The MoS_2_ sample in a custom-made chemical cell filled with Li-TFSI was excited with filtered supercontinuum white light (Fianium WhiteLase) passed through a 532 ± 5 nm band-pass filter and focused onto the flake using a 60×, 1.25 NA oil-immersion objective. (See the Experimental Methods section for details.) The resulting PL was collected through a 660 ± 5 nm (approximately 1.88 ± 0.015 eV) band-pass filter and detected using an electron-multiplying CCD (EMCCD) camera. The 660 ± 5 nm band-pass filter covers A-exciton emission for MoS_2_ (orange region in Figure 1B). The PL peak undergoes a blue shift of approximately 16 meV upon treatment, which is small compared with the detection window (∼30 meV) and thus has only a minor effect on the extracted kinetics.^16^ The images at the top right show PL maps of the same flake before and after treatment measured on the photoluminescence (PL) microscopy setup, both displayed using an identical color scale, and confirm the PL enhancement by Li-TFSI treatment. Figure 1B shows the steady-state PL spectra of the monolayer MoS_2_ before and after Li-TFSI treatment. Following the *in situ* measurement, the sample was collected, rinsed with methanol and IPA, and dried under a flow of N_2_ to remove residual chemicals. To minimize spatial variation in the measurement, the spectra were acquired from approximately the same location on the flake before and after treatment within our positional resolution. The PL intensity of exfoliated MoS_2_ increased by a factor of 38 upon Li-TFSI treatment, consistent with previous reports of Li-TFSI-induced PL enhancement.^14,16^ Figure 1C shows the time-resolved evolution of the integrated PL intensity from the whole field-of-view image during treatment. The emission intensity reaches the saturation plateau within approximately 5 min, which defines the reaction time scale under ambient conditions. The schematic in Figure 1C illustrates the chemical interactions underlying the PL enhancement of MoS_2_. In monolayer MoS_2_, sulfur vacancies are either neutral or negatively charged. Li^+^ ions therefore adsorb at these vacancy sites.^16,18^ This enhances the radiative recombination rate by shifting the excitonic population from trions to a neutral-exciton-dominant regime.

**Figure 1:**
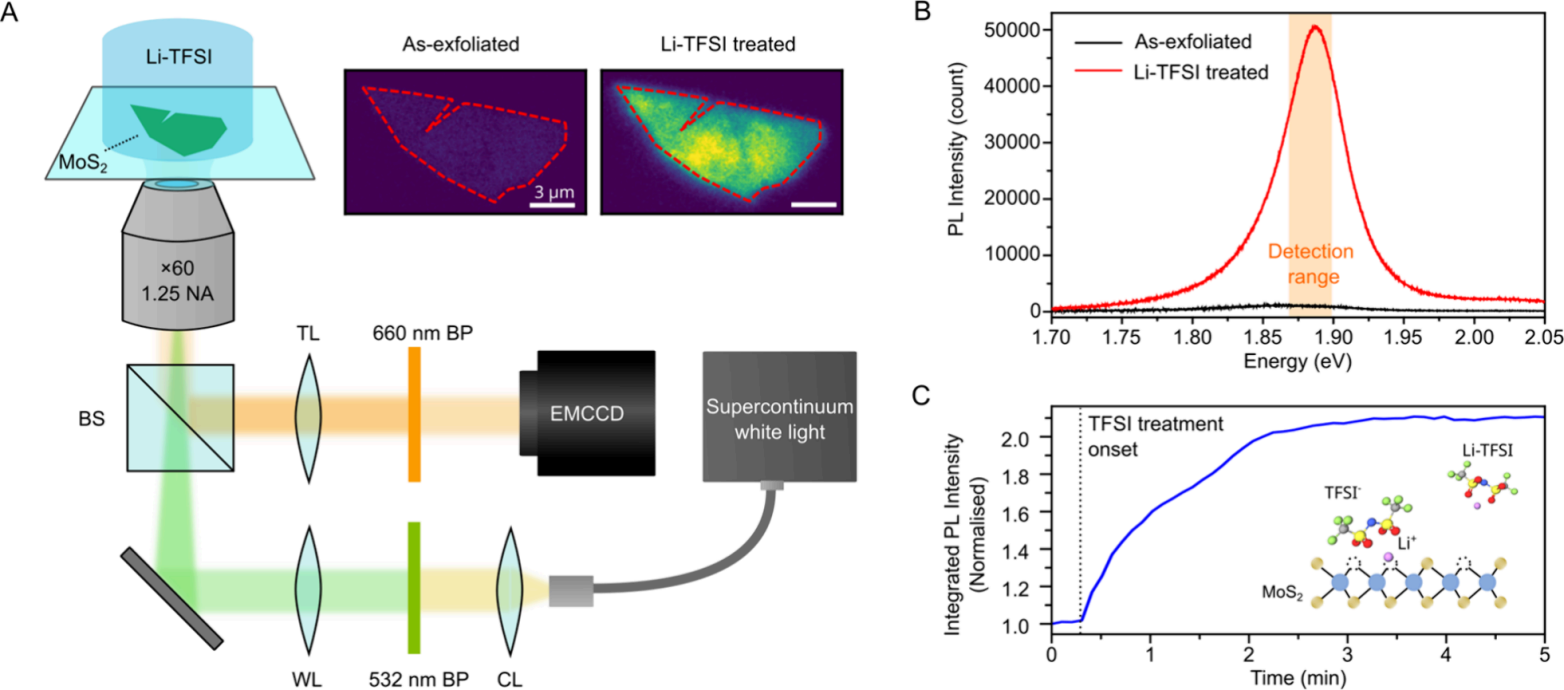
Real-time photoluminescence (PL) monitoring of Li-TFSI treatment on monolayer MoS_2_. (A) Schematic of the home-built PL microscopy setup used for *in situ* monitoring of the PL enhancement of MoS_2_ during Li-TFSI treatment. Upper-right images show PL images of the MoS_2_ flake before and after treatment, displayed on an identical color scale. Scale bar = 3 μm. (B) *Ex* *situ* steady-state PL spectra of monolayer MoS_2_ as-exfoliated (black) and after Li-TFSI treatment, followed by solvent rinsing using methanol and IPA (red). The treated sample exhibits a strong enhancement in neutral-exciton emission. (C) *In* *situ* photoluminescence (PL) enhancement kinetics of monolayer MoS_2_ during treatment. The emission intensity profile exhibits a saturation plateau within approximately 5 min. The schematic illustration on the bottom right presents the chemical treatment mechanism of Li-TFSI on monolayer MoS_2_.

In Figure 2A, we show the evolution of the apparent PL intensity integrated over the monolayer flake (as shown in the red-dotted region in the image in the Figure 1A inset) during in situ Li-TFSI treatment. It increases by approximately 2-fold and reaches saturation after 4.7 min, which is smaller than the 38-fold increase measured *ex* *situ*. The difference in the PL enhancement factor arises due to the inherent differences in the sample and optical geometry compared to the *ex* *situ* measurement, which makes a quantitative comparison difficult (see Methods). Additionally, as we will show later, the actual PL enhancement is much higher than apparent from the raw intensity (ADU) when the dark counts and acquisition parameters are accounted for. The intensity profile indicates that the passivation proceeds on the minutes time scale and, under our concentration and temperature conditions, the characteristic reaction time for Li-TFSI passivation is on the order of 5 min. The PL heterogeneity can be further understood by plotting the distribution of intensities as a histogram from the pixels. This is shown in Figure 2B–E, where the experimental PL intensity histogram (black) is found. The flake PL histogram is calculated by masking the region above the flake (Figure 1A inset, red-dotted region) from the xy-drift stabilized images and extracting the PL intensity per pixel and plotting the intensity distribution as a histogram. This demonstrates the increase in PL intensity, from approximately 1800 ADU at 0.4 min to approximately 3200 ADU at 4.7 min, as well as the broadening of the intensity distribution from approximately 445 ADU to approximately 1540 ADU in the full width at half-maximum (fwhm). However, the fwhm alone is not sufficient to separate the flake-heterogeneity-related broadening with shot-noise and instrumental broadening. In order to elucidate the effect of instrumental- and shot-noise-related broadening, we compare the experimental histogram to the fit from the model (fitting parameters in Table 1). The model assumes a perfectly homogeneous emitter and takes into account the photon-induced signal (*e*_total_), dark noise, read noise, instrument offset, and gain multiplication. (See the Supporting Information for more information.) The model simulates the dark noise histogram accurately (Figure 2B, *R* ^2^ = 0.998, also Figure S1), where the signal comes purely from the dark electrons, gain multiplication, and read noise (*e*_total_ = *e*_dark_). In Figure 2C–E, we account for photons from the sample emission (*e*_total_ = *e*_dark_ + *e*_phot_) and fit the experimental PL histogram to the model. As the model assumes a perfectly homogeneous emitter, a clear mismatch arises from the inherent spatial heterogeneity of the MoS_2_ monolayer. This demonstrates that the broadening shown in the histogram cannot be purely attributed to shot noise and other measurement-related broadening effects. We further explore this in Figure 2F–H, where we present percentile-based PL heterogeneity maps at 0.4, 1.5, and 4.7 min, respectively (indicated as dots in Figure 2A). For each time, pixels belonging to the top 15% (purple), middle 15% (green), and bottom 15% (red) of the PL intensity distribution are overlaid on the flake. At each time, the PL intensity displayed pronounced spatial heterogeneity across the MoS_2_ surface. At early times (Figure 2F, 0.4 min), the lowest- and highest-PL pixels are concentrated near specific regions of the flake, whereas the middle-PL pixels are broadly distributed. The distribution changes significantly as the treatment proceeds (Figure 2G, 1.5 min), where the regions of highest (purple) and lowest (red) intensities shift and become tightly distributed spatially, forming two regions near the interior (highest intensities) or along the top edge and top-right corner (lowest intensities). Interestingly, the spatial distribution of PL heterogeneity does not change significantly after this, even at the end of the enhancement (Figure 2H, 4.7 min), becoming even more tightly distributed across existing regions, demonstrating that spatial heterogeneity remains persistent while the overall PL increases. Beyond 4.7 min, the change in PL intensity plateaus, and the visible change in the spatial heterogeneity is negligible (Figure S2). The redistribution of bright and dark PL regions which spans nearly a micrometer within the first few minutes of treatment cannot be solely attributed to sulfur vacancy migration. Reported activation barriers for sulfur vacancy diffusion in monolayer MoS_2_ range from 0.8 eV to approximately 2.3 eV, depending on the local strain or vacancy density. These values are orders of magnitude larger than the thermal energy at room temperature (*k*_B_ *T* ≈ 0.025 eV), implying that sulfur vacancies are effectively immobile (spatially fixed) under ambient thermal equilibrium.^27,28^ The evolving PL patterns are therefore attributed to the pre-existing disorder pattern on the MoS_2_ flake and the resultant reaction-diffusion kinetics of Li-TFSI on the MoS_2_ surface. While external factors such as substrate-induced doping or strain may contribute to the observed spatial variations,^29^ our findings emphasize that the initially established heterogeneity, whether intrinsic to the MoS_2_ flake or shaped by its environment, serves as a primary template that dictates the subsequent passivation landscape.

**Figure 2:**
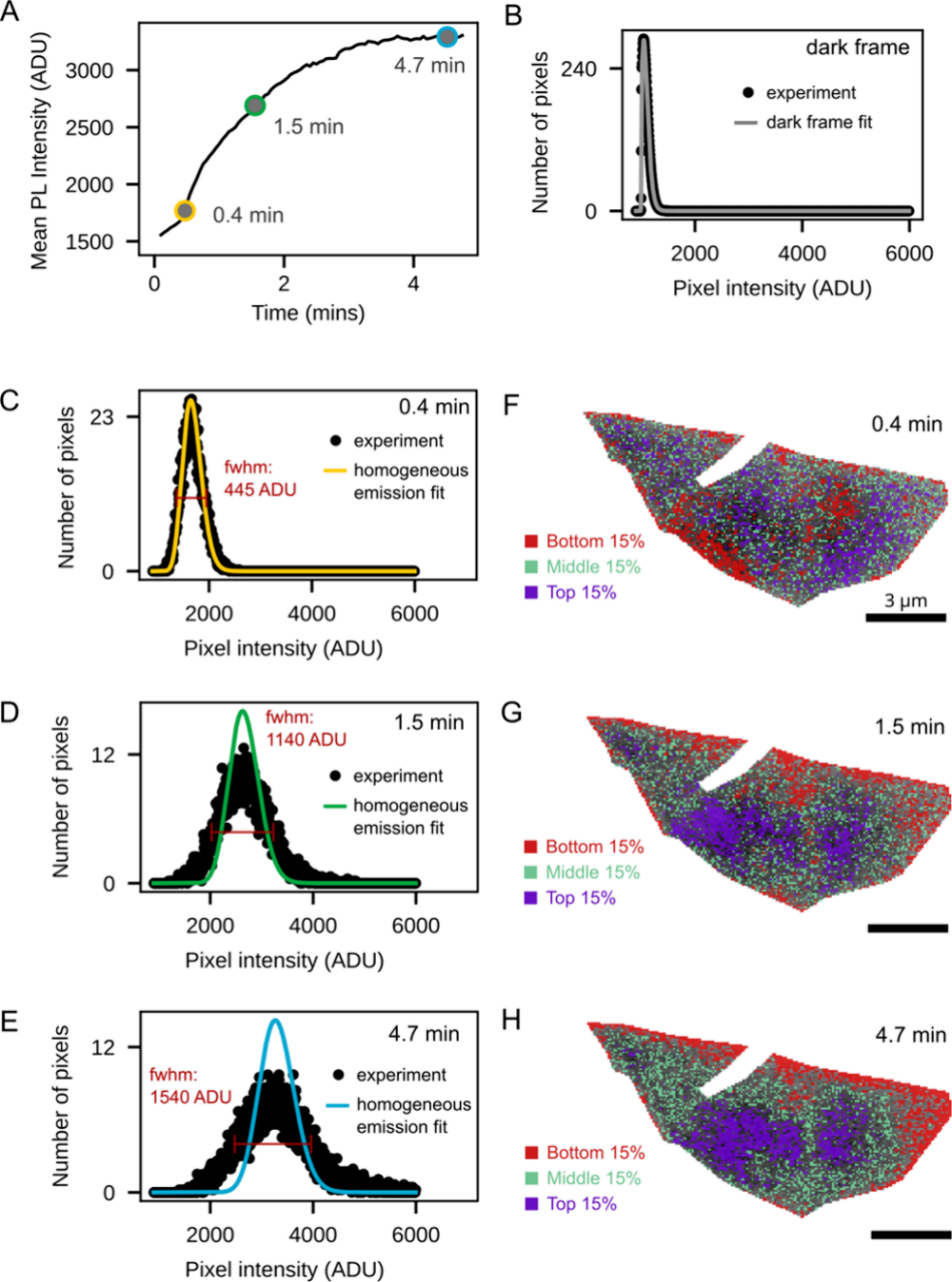
Temporal evolution of PL intensity and spatial heterogeneity. (A) Ensemble PL counts vs chemical treatment duration time. PL counts are averaged over the monolayer area. The time points of interest are marked with a dot. (B–E) PL intensity histogram (black) with the simulated histogram (lines), shown for the dark frame (B) and the PL map taken at various time slices: 0.4 (C), 1.5 (D), and 4.7 min (E). The dark-frame histogram was calculated for 40K pixels, with a bin size of 5 ADU. The PL histogram was calculated for 11562 pixels to be on the flake, with a bin size of 5 ADU. The approximate full-width at half-maximum (fwhm) for the experimental histogram is noted. (F–H) The percentile-based threshold PL map highlights the PL heterogeneity and its time evolution. From the PL map at time slices of 0.4 (F), 1.5 (G), and 4.7 min (H), the pixels with the highest (15% percentile, purple), lowest (15% percentile, red), and middle (15% percentile, green) PL intensities are shown on the flake. Scale bar = 3 μm.

**Table 1:** Fitting Parameters

Conversion factor (ϕ)	ADC offset (*S*_0_)	Gain (G)	Dark noise (e_dark_)	Read noise (σ)
12	1013	300	3.3	75

In Figure 3A, the temporal evolution of the intensity histograms is plotted as a heat map, with the *x* and *y* axes being time and intensity, respectively. Furthermore, the histogram is fitted to the homogeneous emitter model at each time point. The resultant 95% interval from the experiment (black) and from the fitted model (red) is overlaid on the heat map, further demonstrating that the growth in heterogeneity is faster than what is expected from pure shot noise. From the fitted model, the value of *e*_phot_ is plotted against time in Figure 3B. This gives us a good estimate of the actual PL intensity from the flake, revealing the enhancement to be closer to ∼4.5-fold (from ∼19*e*_phot_ to ∼90*e*_phot_). Furthermore, the inherent sample variance can be approximated by subtracting the shot noise and instrumental contribution from the total experimental variance, and it is found to continually increase with this PL intensity enhancement, in agreement with increasing stratification as shown in the spatial distribution shown in Figure 2F–H. Finally, Figure 3D,E shows a schematic of the temporal evolution of the PL enhancement spatial distribution. As expected, most of the spatial distribution and stratification of intensities occur within the first half of the enhancement (by ∼3 min), after which the existing spatial pattern intensifies but does not change significantly spatially. The figure also highlights the discrepancy between the flake interior, which tends to be the brightest, and the edge regions. The stratification of bright vs dark regions is generally observed and supported on other flakes (SI Figure 3). Crucially, the persistent spatial heterogeneity observed throughout the reaction suggests that the final PL distribution of the flake is predetermined by its initial state. This allows forecasting of post-treatment heterogeneity, establishing a foundation for the rational design and improvement of 2D optoelectronic materials. While the present study focuses on mechanically exfoliated MoS_2_ with a defect density of 10^12^–10^13^ cm^–2^, extending this approach to industrially scalable CVD and MOCVD-grown films, which exhibit additional defect modes, will provide further insight into chemical passivation in large-scale 2D materials.^30–32^

**Figure 3:**
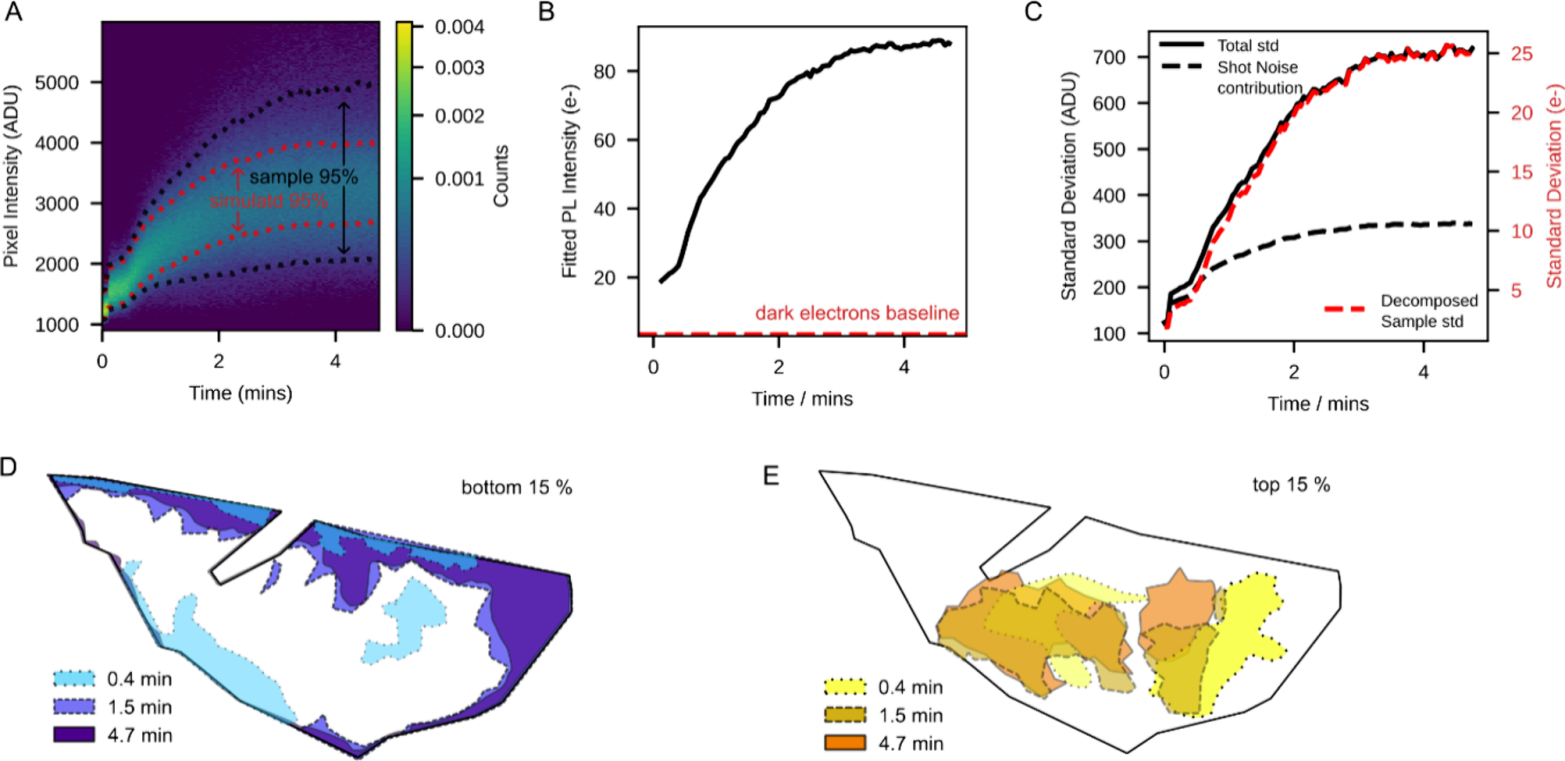
Quantification of PL heterogeneity and spatial evolution of PL intensity in time. (A) PL histogram of MoS_2_ during the chemical treatment, shown as a 2D heat map. The range corresponding to the 95% percentile is shown with the black dotted line. The simulated PL, without any spatial heterogeneity, and its respective 95% percentile range are shown by the red dotted lines. (B) The histogram at every time point is fitted to a homogeneous emitter model, giving the approximate PL intensity as the number of electrons (*e*_photons_). The dark noise baseline (red dashed line, bottom) is constant. (C) Standard deviation from the PL map over time. The total standard deviation from the flake (black solid line, left), the expected noise contribution without spatial heterogeneity (black dashed line, left), and the decomposed standard deviation from the PL intensity are also shown for comparison. (D, E) Schematic showing the spatial extent of the weakest emitting (bottom 15%, D) and strongest emitting (top 15%, E) regions, demonstrating the change over time.

We directly visualized the spatiotemporal evolution of photoluminescence during Li-TFSI passivation of monolayer MoS_2_ with real-time PL microscopy. While we observed a substantial enhancement in ensemble emission, the process remained spatially heterogeneous, with distinct patterns emerging at the onset of the reaction and persisting and becoming more pronounced throughout the treatment. Consistent with the large activation barriers for vacancy migration, these observations indicate that the chemical passivation proceeds along a pre-existing spatial intrinsic defect landscape rather than through defect redistribution. Chemical passivation does not necessarily homogenize the optoelectronic response but instead follows the underlying material disorder and enhances the PL intensity. Our results identify spatial kinetics as a key factor in postsynthetic chemical treatments of two-dimensional semiconductors and suggest that achieving uniform functionality depends on controlling the disorder landscape during material synthesis. The observed spatial kinetics also establish real-time PL imaging as a powerful, nondestructive diagnostic tool for monitoring site-specific charge-transfer dynamics and reaction-diffusion kinetics in 2D semiconductors. Ultimately, this approach establishes real-time PL imaging as a noninvasive diagnostic probe of the underlying disorder within 2D semiconductors and their post-treatment by bridging the gap between atomic-level defects and postsynthetic treatment.

## Experimental Methods

*Mechanical Exfoliation*. MoS_2_ flakes were roughly preexfoliated on blue tape and then transferred onto PDMS tape. Monolayer flakes with suitable size and shape were identified using an optical microscope and transferred onto an ultrathin precision coverslip glass (Thorlab) for oil-immersion microscopy.^19^

*In Situ Li-TFSI Treatment:* *Preparation for Li-TFSI Solvent*

A solution containing Li-TFSI was prepared by dissolving the Li-TFSI powder (Sigma-Aldrich) in methanol at a concentration of 5 mg mL^–1^.

*In Situ Li-TFSI Treatment:* *In Situ Cell Preparation*. The ultrathin precision coverslip with monolayer MoS_2_ was enclosed within a small polypropylene reservoir fabricated from a trimmed pipet tip and sealed to the substrate with epoxy. A narrow opening side was left at the top reservoir to allow reagent delivery. Li-TFSI solution was introduced through this opening using a syringe, whose position was fixed during the measurement to minimize mechanical vibration. Although the reservoir remained partially open, the geometry of the chamber effectively suppressed evaporation, and no noticeable change in the liquid volume was observed until the PL intensity reached saturation.

*In Situ Li-TFSI Treatment:* *Steady-State Optical Characterization*. Steady-state PL measurements were performed under ambient conditions using a Renishaw inVia Raman microscope equipped with a 532 nm excitation laser. Prior to the measurements, the spectrometer was calibrated using a silicon reference sample to account for instrument response and ensure a stable reference peak. During the measurements, the laser power was set to 0.05% (<0.5 μW) and focused onto the specific point of the flake using a 20× long-working-distance objective. The emitted light was collected in streamline mode and dispersed by an 1800 line/mm grating.

*In Situ Li-TFSI Treatment:* *In Situ Acquisition and Analysis*. An EM-CCD (QuantEM 512SC) camera was used to capture a series of 100 images over a 6 s interval, at an integration time of 5000 ms, using a conversion factor of 12 and an EM gain of 300. The MoS_2_ sample was excited at 532 ± 5 nm (Fianium WhiteLase) and imaged using the same objective (60×, 1.25 NA) via epi-illumination geometry. The data was stored as a numpy array and analyzed via a custom python script. Note that the differences in experimental conditions and optical configuration between the in situ and ex situ measurements make it very difficult to compare the PL enhancement factor. For example, in an in situ measurement, the dielectric environment of the flake changes from glass/MoS_2_/air (at *t* = 0) to glass/MoS_2_/methanol (Li-TFSI solution), changing the effective absorption of the flake even under constant illumination. Furthermore, for the in situ analysis, the PL is integrated over the entire flake, whereas the ex situ measurement is usually taken from the same spot in the interior of the flake.

To analyze the particle PL intensities, the initial image stack was first stabilized for xy-drift manually and then the pixels inside the particle were extracted using a masked region of interest and stored as a sparse array for further analyses. A time-dependent histogram of these values was calculated using a bin size of 5 ADUs and normalized. The experimental distribution was least-squares fitted to an analytical EMCCD model as previously described.^33^ Parameters used for fitting can be found in Table 1, and detailed modeling and fitting procedures can be found in the Supporting Information.

## Supplementary Material



## Notes

Detailed description of the EMCCD noise model; dark frame histogram fitting (Figure S1); extended time series of percentile-based PL threshold maps from 0.1 to 9.0 min (Figure S2); and PL threshold maps from additional MoS_2_ flakes confirming consistent spatial heterogeneity patterns (Figure S3) (PDF)
