# Supplementary material for: Nonuniform Chemical Passivation Dynamics in Monolayer MoS2 from Real-Time Photoluminescence Imaging
Source: J Phys Chem Lett. 2026 May 22;17(22):6317–22. doi: 10.1021/acs.jpclett.6c00839 (PMC13244547; doi:10.1021/acs.jpclett.6c00839)
Supplement: Supplementary file 1 [file jz6c00839_si_001.pdf]

Supplementary information for

Nonuniform Chemical Passivation Dynamics in Monolayer MoS_2 from Real-Time Photoluminescence Imaging

Juhwan Lim[1,2,†], Jiho Han[1,†], Nicolas Gauriot[1], Zhaojun Li[1,3], Jung-In Lee[2], Christoph Schnedermann[1], Manish Chhowalla[2*], Akshay Rao[1*]

[1]Cavendish Laboratory, University of Cambridge, JJ Thomson Avenue, Cambridge CB3 0HE, United Kingdom

[2]Department of Materials Science and Metallurgy, University of Cambridge, 27 Charles Babbage Road, Cambridge CB3 0FS, United Kingdom

[3]Solid State Physics, Department of Materials Science and Engineering, Uppsala University, P.O. Box 35 Uppsala, Sweden

[†]Juhwan Lim and Jiho Han equally contributed to this work

*E-mail: mc209@cam.ac.uk, ar525@cam.ac.uk

Supplementary Methods:

In brief, the photoluminescence (PL) from the flake is collected by the sensor, generating photoelectrons via a Poisson process $\mathrm{P}(\lambda)$, where λ is the total number of photoelectrons or e_{total}. This is compounded with the EM multiplication process, modeled by a Gamma distribution $\Gamma(k, G)$ with EM gain G. The read noise is normally distributed $\mathrm{N}(0,\, \sigma^2_{read})$. This yields a Poisson-compounded Gamma (PG) distribution for the former:

$$f_Y(x) = \sqrt{\frac{\lambda}{Gx}} \exp\left(-\lambda - \frac{x}{G}\right) I_1\left(2\sqrt{\frac{\lambda x}{G}}\right)$$

and a normal distribution for the latter:

$$f_X(x) = \frac{1}{\sqrt{2\pi}\sigma_{\text{read}}} \exp\left(-\frac{x^2}{2\sigma^2_{\text{read}}}\right)$$

The complete probability density function is computed via Fast Fourier Transform (FFT) convolution in the electron domain. The model can be aligned with the experimental data, by first mapping from the ADU domain to the electron domain using $\mathrm{x} = \phi \times (S - S_0)$, where S is the raw signal in ADU, S_0 is the camera bias offset, and ϕ is the conversion factor (e$^-$/ADU). Subsequently, the resulting PDF is scaled back to the ADU domain by multiplying by ϕ to preserve the total probability.

The experimental histogram was fitted to the analytical model in python using scipy.optimise.least_squares with default tolerances. The conversion factor (ϕ), ADC offset (S_0), and gain (G) were known. Initially, dark noise (e_{dark}) and read noise (σ_{read}) were found by dark frame fitting assuming that the $\lambda = e_{total} = e_{dark}$ as $e_{photon} = 0$, and assumed to be constant for subsequent fitting. This allows us to find the best fit (λ) for the experimental histogram from the flake for each frame, giving an approximate e_{photon} under a homogeneous emitter assumption as discussed in the main text.

Supplementary 1.

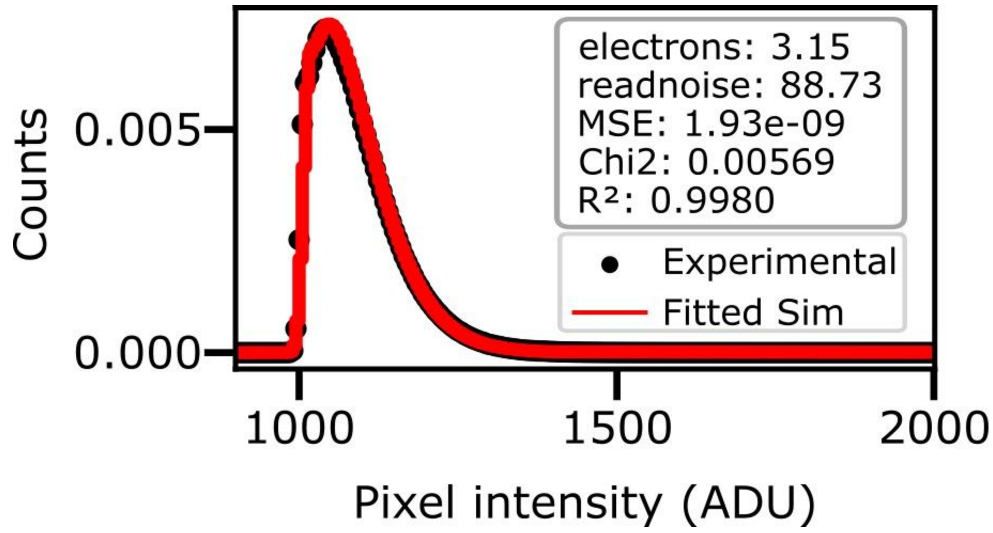

Figure S1. Dark noise histogram

Supplementary 2.

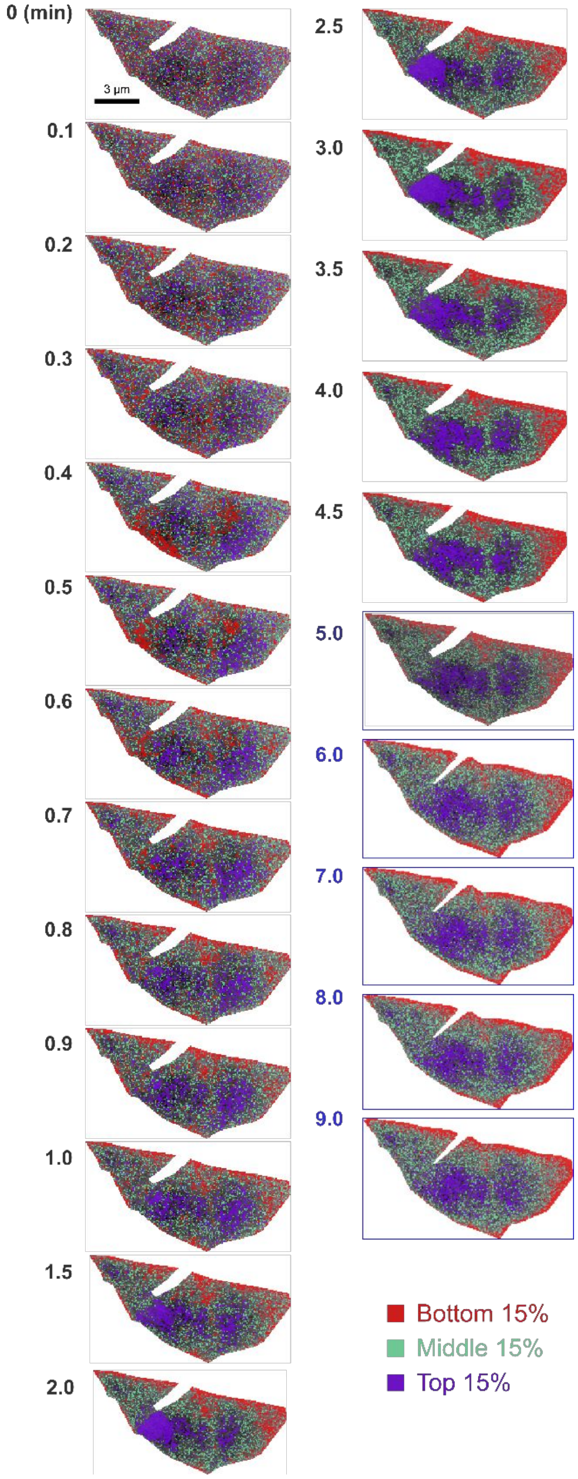

Figure S2. Temporal evolution of PL intensity and spatial heterogeneity. Percentile-based threshold PL map highlights the PL heterogeneity and its time evolution. PL map at time slices from 0.1 min to 9.0 min. The pixels with the highest (15% percentile, purple), lowest (15% percentile, red) and middle (15% percentile, green) PL intensities, are shown on the flake. Scale bar = 3 μm.

Supplementary 3.

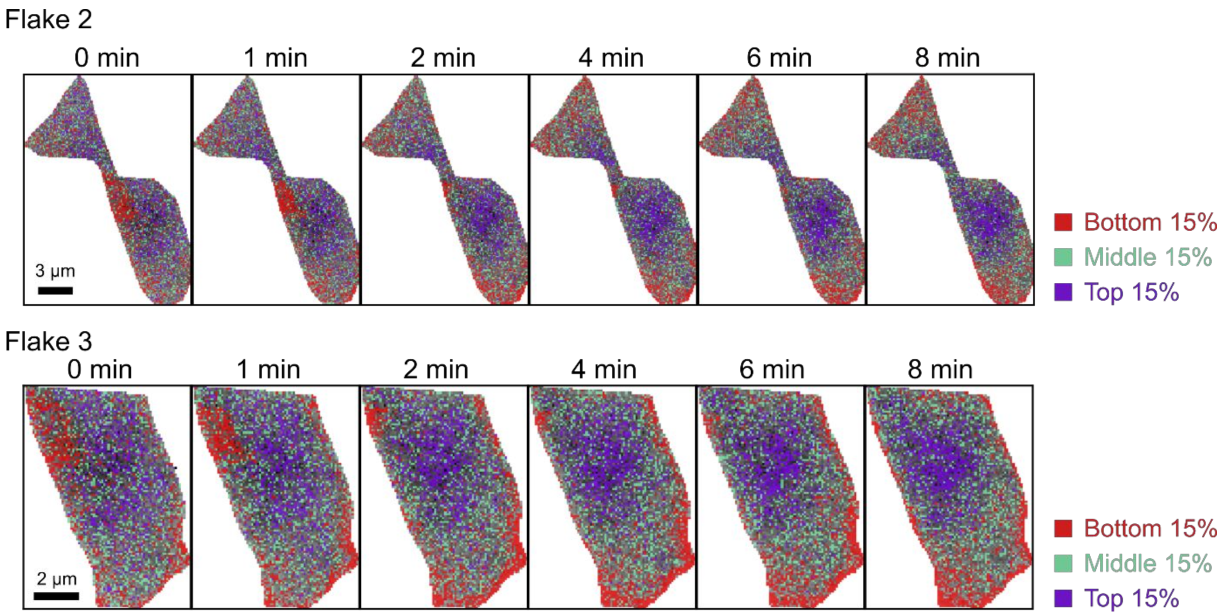

Figure S3. PL threshold map from different flake. TFSI-based PL enhancement begins at 0 min. Scale bar = 3 µm for flake 2, Scale bar = 2 µm for flake 3.
